# Resignation of the Post-Graduate Faculty of the Medical Department of the University of the City of New York

**Published:** 1882-05

**Authors:** 


Resignation of the Post-graduate Faculty of the Medical Department of the University of the City of New York.—On account of differences between the governing body and the post-graduate faculty of the University Medical School, the following members of the latter body have tendered their resignations, which were accepted: Professors D. B. St. John Roosa, Wm. A. Hammond, J. W. S. Gouley, Montrose A. Pallen, Frederic R. Sturgis, H. G. Piffard, Stephen Smith and J. L. Little. It is rumored that these gentlemen, with others, propose to organize an endowed school, for post-graduate and clinical instruction merely, to be attached to Cornell University as its medical department. The medical school will, of course, be located in this city.

